# Measles vaccination: A matter of confidence and commitment

**DOI:** 10.1371/journal.pmed.1002770

**Published:** 2019-03-26

**Authors:** Richard Turner, on behalf of the PLOS Medicine Editors

**Affiliations:** Public Library of Science, San Francisco, California, United States of America, and Cambridge, United Kingdom

## Abstract

The *PLOS Medicine* Editors discuss issues of vaccination uptake in the context of recent and ongoing measles outbreaks.

Prominent among the anxieties of our times are those regarding health and disease. Not only are ageing populations expected to suffer an increased burden of noncommunicable diseases in the future, but risks of and harms from existing and emerging infectious diseases could also increase, owing to population growth, migration, climate change, and other factors. At the population level, it is clear that the hard-won gains in medicine and public health brought about by vaccination, antimicrobial and other treatments, and improved sanitation will need to be sustained, broadened, and intensified to protect and provide for an increasing proportion of the human population. Global aspirations, including those set out in the Sustainable Development Goals, are unambiguous in setting out an anticipated future trajectory of improved health, well-being, and prosperity.

Measles, a highly contagious viral infection, is in various respects the perfect example of a threat to health that respects neither aspirations nor boundaries between developed and developing countries. Complications of measles infection include pneumonia (the most common cause of death in children with measles), encephalitis, ear infections that can lead to permanent deafness, and diarrhoea. Although a safe and very effective two-dose vaccination schedule has been available and widely deployed since the 1960s, the need for very high and consistent vaccination coverage to elicit herd immunity means that the disease burden and harms of measles remain substantial. WHO reports that globally, in an apparent uptrend of cases occurring in 2017, measles led to an estimated 110,000 deaths, most in children aged under 5 years [1]. Tragically, these deaths were unavoidable, given the estimated 20.8 million children in low- and middle-income countries who had not received a single dose of measles vaccine through routine programmes in that year.

In the 53 high- and middle-income countries that make up its European region, WHO has indicated that around 82,500 cases of measles were reported in 2018, an alarmingly high number even among a population in excess of 900 million people, and a greater than 3-fold increase since 2017 [2]. There were 72 reported deaths in children and adults. Here, the European Vaccine Action Plan 2015–2020 recognizes the need for high and consistent levels of vaccine coverage yet acknowledges the difficulties in meeting the challenges of achieving high and equitable coverage, owing to the existing pronounced variations in national and regional coverage with measles vaccination.

In the United States, despite the declared elimination of measles in 2000, outbreaks have been well documented in recent years—the outbreak in Southern California during December 2014–February 2015 involved at least 125 cases [3]. Among these cases, a substantial proportion were in people known not to have been vaccinated, including infants who were too young to have been protected and individuals who had chosen not to receive measles vaccination (i.e., 49 people were unvaccinated among the 110 cases identified in California). More recently, an outbreak in Clark County, Washington State has been widely reported in the past few weeks, and at the time of writing there had been 65 confirmed measles cases in this area [4]. In 2018, writing in *PLOS Medicine*, Jacqueline Olive and colleagues highlighted clusters of people claiming nonmedical exemptions from measles vaccination in states where this is permitted by law [5]. The authors noted that ‘new foci of antivaccine activities are being established in major metropolitan areas, rendering select cities vulnerable for vaccination-preventable diseases.’ It is difficult to imagine a future scenario without repeated and serious measles outbreaks in the US and elsewhere, given the gaps in protection against the disease. A cautionary indication of the extent to which the dangers of so-called ‘vaccine hesitancy’ can escalate is in the Philippines, where there are reported to have been thousands of measles cases and at least 189 deaths since the beginning of 2019, mainly in children [6].

The reasons for people not accepting vaccination against measles and other potentially fatal and readily preventable infections are, unfortunately, all too well known. Fears about potential harms of the combined measles, mumps, and rubella vaccination were raised by a discredited study published in *The Lancet* in 1998 and are continuing to circulate. As Peter Hotez, Dean of the National School of Tropical Medicine at Baylor College of Medicine, Houston, Texas, commented to *PLOS Medicine*, ‘the “anti-Vax” movement began as a fringe group but has now become a media empire in its own right, producing hundreds of websites, books, and videos. Even if a concerted effort were mounted against this movement, it could take years to be effective.’ Despite the volumes of scientific research and debate published in the intervening 20 years, supporting beyond reasonable doubt the overwhelmingly favourable benefit:risk assessment for vaccination against measles and other infectious diseases, levels of scepticism clearly persist and are being propagated in susceptible populations worldwide. It seems that the growth of social media has facilitated the development of geographically widespread communities with fixed yet indefensible opinions, where hearsay is spread intensively while robust medical evidence and guidance hold little sway.

It would probably be unwise to expect a single approach or constituency to be able to change minds opposed to vaccination. In the case of measles, high-quality surveillance activities alongside well-supported and planned vaccination programmes are essential to bring about progressive reductions in the high burden of morbidity and deaths in developing countries. In settings where limited public acceptance of measles vaccination is a danger, imaginative governmental and, where viable, civil society- or NGO-led information campaigns are needed to drive uptake of vaccination, alongside the essential underpinnings of culturally appropriate incentives and legal provisions. Healthcare professionals, who in many countries are generally trusted and enjoy a high level of confidence from the public, are likely to be an underused resource in conveying accurate information and advice on vaccines and vaccination through formal and informal routes. Ultimately, the question is one of altruism: everyone who has experienced the silent but long-lasting protection afforded by vaccination has the responsibility to promote understanding and acceptance of the benefits to others. Our neighbours and, most of all, their children, deserve nothing less.
